# Accuracy of Periapical Radiography and CBCT in Endodontic Evaluation

**DOI:** 10.1155/2018/2514243

**Published:** 2018-10-16

**Authors:** R. Lo Giudice, F. Nicita, F. Puleio, A. Alibrandi, G. Cervino, A. S. Lizio, G. Pantaleo

**Affiliations:** ^1^Department of Clinical and Experimental Medicine, Messina University, Policlinico G. Martino, Messina, Italy; ^2^Department of Biomedical and Dental Sciences and Morphofunctional Imaging, Messina University, Messina, Italy; ^3^Department of Economics, Section of Statistical and Mathematical Sciences, Messina University, Messina, Italy; ^4^Department of Neurosciences, Reproductive and Odontostomatological Sciences, Naples Federico II University, Naples, Italy

## Abstract

**Introduction:**

A radiological evaluation is essential in endodontics, for diagnostic purposes, planning and execution of the treatment, and evaluation of the success of therapy. The periapical radiography is nowadays the main radiographic investigations used but presents some limits as 3D anatomic alteration, geometric compression, and possible anatomical structures overlapping that can obscure the area of interest. CBCT (cone beam computed tomography) in endodontics allows a detailed assessment of the teeth and surrounding alveolar anatomy for endodontic diagnosis, treatment planning, and follow-up.

**Objective:**

The purpose of this study was to evaluate the accuracy of CBCT in comparison with conventional intraoral radiographs used in endodontic procedures.

**Materials and Methods:**

Statistical analysis was performed on 101 patients with previous endodontic treatments with the relative radiographic documentation (preoperative, postoperative, and follow-up intraoral X-ray) that had underwent at CBCT screening for surgical reasons. The CBCT scans were evaluated independently by two operators and compared with the corresponding periapical images.

**Results:**

Our analysis shows that the two radiological investigations statistically agree in 100% of cases in the group of patients without any endodontic sign. In the group of patients with an endodontic pathology, detected with CBCT, endodontic under extended treatments (30.6%), MB2 canals in nontreated maxillary molars (20.7%), second canals in nontreated mandibular incisors (9%), root fractures (2.7%), and root resorption (2.7%) were not always visible in intraoral X-ray. Otherwise, positivity in the intraoral X-ray was always confirmed in CBCT. A radiolucent area was detected in CBCT exam in 46%, while the intraoral X-ray exam was positive only in 18%.

**Conclusions:**

Our study shows that some important radiological signs acquired using CBCT are not always visible in periapical X-ray. Furthermore, CBCT is considered as a II level exam and could be used to solve diagnostic questions, essential to a proper management of the endodontic problems.

## 1. Introduction

Radiology is essential in endodontics for diagnostic purposes, planning and execution of the treatment, and evaluation of the success of therapy [1].

Until few years ago, the main radiographic investigations used in the endodontic treatment were periapical radiography and, for a general evaluation, orthopantomography.

The conventional radiographic techniques show some limits. These include the following:

- *Anatomic 3D compression*. The conventional radiography gives a two-dimensional image, obliging the operator to perform many X-rays with different projections in numerous cases in order to obtain a complete display of the teeth and nearby tissues anatomy [2, 3].
- *Geometric alteration*. For an accurate anatomy reproduction, the image receptor should be parallel to the longitudinal tooth axis and the radiogenic font perpendicular to them. An overangulated or downangulated radiography reduces or increases the roots' length and the tooth dimension, and it can determine diagnostic omissions of periradicular pathologies [4–6]. The distortion degree of the anatomic structures could range from 3.4% for the periapical radiography to more than 14% for OPT (orthopantomography) [7].
- *Anatomic obstacles*. Some anatomic structures can obscure the area of interest causing a difficult radiological interpretation of the images [8]. So, in the routine clinical practice, there are some cases in which the conventional radiography does not give sufficient information on the pathological conditions, anatomic shapes of the structures, and positional relations.

Ex vivo and in vivo studies confirm that two-dimensional radiology presents clear limits in the periapical lesion diagnoses [9, 10].

One of the factors that highly influence the lesion recognition is bone thickness. Indeed, it has been established that, in an intraoral radiogram, the lesions which involve only the bone medullary component may pass unobserved because of the overhead cortical lines up to the radiolucent area [11–13].

Moreover, two-dimensional images sometimes do not allow to detectthe real number of root canals with consequences on the success rate [14, 15].

The modern systems of digital radiographic imaging introduced relevant improvements in endodontics. The quality of the image is highly important in endodontics because it makes easier the accurate interpretation of the endodontic anatomy, and in particular, the detection of possible canal curvatures, as well as the postoperatory evaluation and long-term result of the endodontic treatment [16–18].

The CBCT permitted a detailed three-dimensional evaluation of the teeth, maxillofacial skeletal district, and relation among anatomical structures [19, 20].

The CBCT in endodontics not only gives a three-dimensional evaluation of the region of interest but also an appropriate resolution of images that allows a detailed analysis of tooth and surrounding alveolar anatomy.

The guidelines of the European Society of Endodontology suggest the use of CBCT in endodontics in limited cases as follows [21]:

- Periapical pathology diagnosis in presence of contradictory (not specific) signs and/or symptoms
- To confirm the causes of nonodontogenic pathology
- Maxillofacial trauma evaluation and/or treatment quality
- The extremely complex root canal anatomy evaluation before endodontic orthograde retreatment
- The evaluation of the causes of the endodontic failure in surgical endodontic treatment planning
- Evaluation and/or management of radicular resorption.

Therefore, CBCT can be a powerful instrument in endodontic diagnosis, as well as in the treatment planning and follow-up.

At the same time, the decision to expose a patient to a CBCT investigation must be done evaluating risk/benefit ratio in each case, which is determined by the necessity to obtain the optimal endodontic treatment management [22, 23].

The purpose of this study is to compare the accuracy of CBCT imaging with periapical radiographs in the interpretation of clinical endodontic situations.

## 2. Materials and Methods

### 2.1. Patient Selection

Our research has been conducted on patients treated between 2015 and 2018 in the Department of Dentistry of Messina University. The selection was performed according to the following inclusion criteria:

1. Execution of three-dimensional X-ray examination (CBCT) for surgical reasons
2. Presence of at least one tooth previously endodontically treated, with the relative radiographic documentation (pre- and post-operative intraoral X-ray and the follow-up X-ray between 3 and 6 months)
3. Radiographic quality of the images adequate for the evaluation of the periapical status of the teeth.

One hundred and one patients satisfied these criteria and have been submitted for further evaluation.

The CBCT images have been done by using an extraoral radiographic hybrid system (MyRay Hyperion X9 Pan/Ceph/CBCT Scanner).

The equipment accomplishes the reconstruction of three-dimensional mold of the volume examined.

Then, the image is transferred to a computer real time and visualized and saved with the iRYS Software.

### 2.2. Radiographic Evaluation

All the images have been endodontically evaluated separately, by two operators selected as experienced endodontists with more than 10 years of clinical practice and II level master in Endodontics, not directly involved in the patients' treatment planning.

The operators have analyzed each tooth and the periapical structures, highlighting all the images with possible endodontic relevance.

For the CBCT images, the radiolucency should be visible at least in two image plans (0.5 mm thickness).

The CBCT scans have been compared to the corresponding intraoral control X-ray.

For each detected periapical lesion, we evaluated for the following:

1. Under extended endodontic treatments
2. Nontreated canals (MB2 canal in maxillary molars and lingual canal in mandibular incisors)
3. Root fractures
4. Resorptions.

### 2.3. Statistical Analysis

The selected patients were divided into two groups:

- Patients without endodontic pathology in the radiographic documentation at the end of the endodontic treatment and in CBCT
- Patients with an endodontic pathology in intraoral X-ray and/or CBCT.

All the data have been evaluated through preliminary descriptive analysis.

The clinical-statistical evaluations were relevant to the following:

- Absence of lesion in CBCT, Absence of lesion in Rx
- Presence of lesion in CBCT, Absence of lesion in X-ray
- Presence of lesion in CBCT, Presence of lesion in X-ray
- Absence of lesion in CBCT, Presence of lesion in X-ray.

Presence/absence of a periapical radiolucent area and diagnostic concordance between periapical X-ray and CBCT, considering the following the four possible combinations.

The presence of an endodontic pathology or incorrect treatment associated with a periapical radiolucency and the incidence of the diagnostic investigation on the detection of individual clinical situations.

The chi-square test was performed to compare the accuracy of intraoral radiographs and CBCT scans in the detection of periapical lesions and/or endodontic pathologies.

To evaluate diagnostic matching degree between the two instrumental exams, Cohen's kappa coefficient was considered with the following values [24]:

- ≤0.2: bad
- 0.21–0.4: sufficient
- 0.41–0.6: not bad
- 0.61–0.8: good
- 0.81–1: excellent.

The statistical analysis was conducted by using SPSS 17.0 for Windows operating system. A *P* value <0.05 was considered statistically significant.

## 3. Results

The statistical analysis of 111 periapical radiographic images and CBCT showed that signs of endodontic relevance were not present in 34.2% (group A #38). In 65.8% of cases, these signs were observed in the radiological diagnosis exams (group B #73).

**Figure 1:**
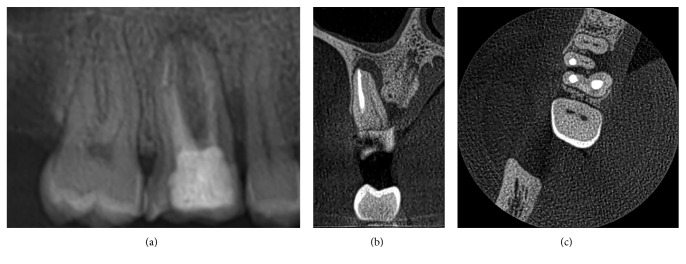
Endodontic treatment 1.6. (a) Periapical X-ray: apex endodontic treatment and periapical radiolucency. (b) CBCT  sagittal section: apex endodontic treatment MB, untreated MB2, and periapical radiolucency. (c) CBCT transversal section: untreated MB2.

In particular, the following diagnostic elements were identified (Table 1):

- 34 cases of endodontic under extended treatments (30.6%)
- 23 cases of MB2 canals nontreated maxillary molars (20.7%) (Figure 1)
- 10 cases of second canals nontreated mandibular incisors (9%)
- 3 cases of root fractures (2.7%)
- 3 cases of internal or external root resorption (2.7%).

**Figure 2:**
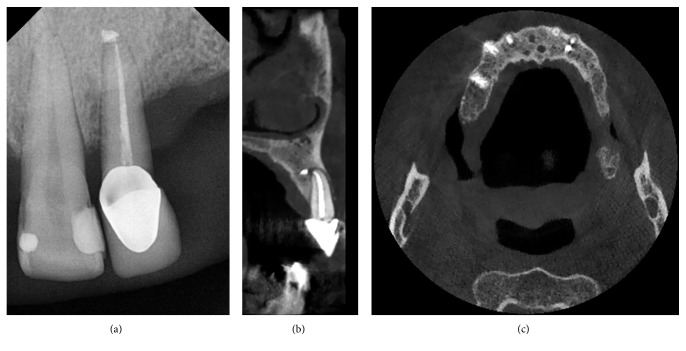
Endodontic treatment 2.2. (a) Periapical X-ray: endodontic overfilling and no periapical radiolucency. (b) CBCT sagittal cross section: apex endodontic treatment, over filling, and periapical radiolucency. (c) CBCT transversal section: over filling and periapical radiolucency.

In group B, 70% of the cases had developed a periapical lesion. The radiolucent area was found in the CBCT exam in 51 cases on 111 (46%), while the endoral X-ray exam was positive only in 20 cases (18%) (Figure 2).

The prevalence of endodontic under extended therapy in the context of the examined trends is 34 cases on 111 (30.06%). In the 100% of cases, there was diagnostic agreement between endoral X-ray and CBCT.

The chi-square test reveals the existence of a perfect statistic concordance between the two diagnostic exams. The K Cohen's coefficient highlights an excellent agreement (1000) among the surveys performed by using endoral X-ray and CBCT.

The distribution of periapical radiolucency detected in association with the correspondent endodontic pathology is summarized in Table 2.

The chi-square test highlights a significant association between the two diagnostic exams in detecting the presence of radiolucent area and under extended endodontic treatments. Moreover, the K Cohen coefficient reports the values 0.411 and 1000, respectively, for periapical lesion and underextended treatments. The data obtained from the two analysis performed are described in Table 3.

## 4. Discussion

The presence of an apical periodontitis represents an important prognostic factor [25, 26].

However, it was demonstrated that periapical lesions are visible on radiography only when the periapical pathology determines a 30%–50% loss of bone structure [27].

The intraoral images technique shows many evident limitations related to a bidimensional representation of three-dimensional structures and often gives insufficient information about the dimension, extension, and position of the periapical lesion [2].

Nowadays, the intraoral examination represents the routine investigation for the diagnosis formulation, the planning of treatment, and the evaluation of success [28].

The introduction of cone beam computed tomography (CBCT) scanning determined important advantages for the diagnosis of endodontic pathology.

Our descriptive analysis shows that the two radiological investigations (CBCT and intraoral X-ray) agree in 100% of cases in the group of patients without any endodontic sign (A group).

However, the presence of an endodontic pathology or an incorrect treatment, associated or not to a periapical radiolucency, was not always visible in intraoral X-ray.

On the contrary, positivity in the periapical X-ray was always detectable in CBCT. This fact is confirmed by recent studies which showed how CBCT gives more accurate information in the survey of endodontic signs [29–31], avoiding anatomic structure overlapping [32, 33].

Our research points out that the periapical lesions detected in the context of all the examined CBCT scans are 51 cases. Only in 20 cases the diagnostic agreement was recorded between the two instrumental exams. Even Cheung et al. and Venskutonis et al. reported, respectively, an improvement of 63% and of 57.1% on the periapical lesions quality detection with CBCT [31, 34].

In addition, Cohen's kappa coefficient shows a decent agreement between the endoral X-ray and CBCT surveys, in spite of a relevant percentage of diagnostic discordance (27.9%).

Therefore, although CBCT is obviously more reliable in identifying signs of endodontic relevance than conventional radiography, the latter retains an effective validity.

In vitro studies have shown the greater reliability of CBCT images compared to conventional endoral X-ray in the pathology diagnosis of endodontic relevance such as root fracture, root perforation, and resorption [8, 30, 35, 36].

Our study highlights that, only in CBCT, scans are detected: root fractures (2.7%) and resorption (2.7%).

Regarding the iatrogenic errors, we have noticed the missing treatment of MB2 (20.7%) and the lingual canal of the lower incisors (9%).

In case of underextended endodontic therapies, there is a total diagnostic agreement between endoral X-ray and CBCT.

Our analysis shows that endodontic underextended treatments are more frequently associated with a periapical lesion than other endodontic diseases (31 out of 51 cases). Furthermore, some radiolucent lesions associated with no treatment of MB2 and/or mandibular incisors' lingual canal are also evident in the X-ray, despite the presence of the untreated canals which has been ascertained only in CBCT scans.

## 5. Conclusions

Our research shows that many of the endodontic signs obtained from the analysis of CBCT images are not resulted in the corresponding intraoral radiographs. The use of two-dimensional radiology therefore shows clear limits that can be overcome by 3D examinations.

Cone beam is therefore indispensable in all those cases in which a discrepancy between the clinical examination and the diagnostic evidence that can be objected to the intraoral radiographic examination is observable.

To perform a 3D examination, it is essential that the radiation dose is kept “at the lowest level reasonably obtainable” and that the FOV is limited only to the region of interest [37, 38].

However, the use of intraoral radiographs in different projections may increase the possibility of a correct diagnosis compared to a single radiograph.

Consequently, the CBCT remains a second level survey to be used adequately exploiting the system potential (correct FOV settings, mAs, appropriate kVp, and selection of the definition parameters) according to the ALADA concept (dosage as low as acceptable from the point of diagnostic view).

## Figures and Tables

**Table 1:** Comparison of diagnostic evidences detection between CBCT and intraoral X-ray.

Diagnostic evidences	CBCT	Intraoral Rx	Total (%)
Root fractures	3	/	2.7
Underextended endodontic treatments	34	34	30.6
Internal/external root reabsorption	3	/	2.7
Lack of superior molar's MB2 treatment	23	/	20.7
Lack of a inferior incisor's lingual canal	10	/	9

**Table 2:** Comparison periapical lesions detection related to different endodontic and iatrogenic pathologies.

Diagnostic evidences	Periapical lesions (CBCT)	Periapical lesions (X-ray)
Under extended endodontic treatment	31	11
Nontreated MB2 canals	11	5
Nontreated lingual canals	7	4
Root fractures	1	—
Int/ext reabsorption	1	—
Total	51	20

**Table 3:** Statistical analysis: association between CBCT and intraoral Rx results in diagnosis of radiolucent areas and underextended treatments.

	Pearson's chi-square	Cohen's kappa
Radiolucent area	28.701/0.000	0.411/0.000
Under-extended treatments	111.000/0.000	1/0.000

## Data Availability

The data used to support the findings of this study are available from the corresponding author upon request.

## References

[1] Reit C., Petersson K., Molven O. (2003). *Diagnosis of Pulpal and Periradicular Disease. Textbook of Endodontology, *.

[2] Velvart P., Hecker H., Tillinger G. (2001). Detection of the apical lesion and the mandibular canal in conventional radiography and computed tomography. *Oral Surgery, Oral Medicine, Oral Pathology, Oral Radiology, and Endodontology*.

[3] Lo Giudice G., Nigrone V., Longo A., Cicciù M. (2008). Supernumerary and supplemental teeth: case report. *European Journal of Paediatric Dentistry*.

[4] Forsberg J., Halse A. (1994). Radiographic simulation of a periapical lesion comparing the paralleling and the bisecting-angle techniques. *International Endodontic Journal*.

[5] Lo Giudice G., Lipari F., Lizio A., Cervino G., Cicciù M. (2012). Tooth fragment reattachment technique on a pluri traumatized tooth. *Journal of Conservative Dentistry*.

[6] Lo Giudice G., Alibrandi A., Lipari F. (2017). The coronal tooth fractures: preliminary evaluation of a three-year follow-up of the anterior teeth direct fragment reattachment technique without additional preparation. *Open Dentistry Journal*.

[7] Lazzerini F., Minorati D., Nessi R., Gagliani M., Uslenghi C. M. (1996). The measurement parameters in dental radiography: a comparison between traditional and digital techniques. *La Radiologia Medica*.

[8] Paurazas S. B., Geist J. R., Pink F. E., Hoen M. M., Steiman H. R. (2000). Comparison of diagnostic accuracy of digital imaging by using CCD and CMOS-APS sensors with E-speed film in the detection of periapical bony lesions. *Oral Surgery, Oral Medicine, Oral Pathology, Oral Radiology, and Endodontology*.

[9] Jorge E. G., Tanomaru-Filho M., Gonvalves M., Tanomaru J. M. (2008). Detection of periapical lesion development by conventional radiography or computed tomography. *Oral Surgery, Oral Medicine, Oral Pathology, Oral Radiology, and Endodontology*.

[10] Paula-Silva F. W. G., Wu M.-K., Leonardo M. R., da Silva L. A. B., Wesselink P. R. (2009). Accuracy of periapical radiography and cone beam computed tomography in diagnosing apical periodontitis using histopathological findings as a gold standard. *Journal of Endodontics*.

[11] Patel S., Dawood A., Mannocci F., Wilson R., Pitt Ford T. (2009). Detection of periapical bone defects in human jaws using cone beam computed tomography and intraoral radiography. *International Endodontic Journal*.

[12] Tsai P., Torabinejad M., Rice D., Azevedo B. (2012). Accuracy of cone-beam computed tomography and periapical radiography in detecting small periapical lesions. *Journal of Endodontics*.

[13] Gao Y., Haapasalo M., Shen Y., Wu H., Jiang H., Zhou X. (2010). Development of virtual simulation platform for investigation of the radiographic features of periapical bone lesion. *Journal of Endodontics*.

[14] Patel S. (2009). New dimensions in endodontic imaging: part 2. Cone beam computed tomography. *International Endodontic Journal*.

[15] Zheng Q.-H., Wang Y., Zhou X.-D., Wang Q., Zheng G.-N., Huang D.-M. (2010). A cone-beam computed tomography study of maxillary first permanent molar root and canal morphology in a Chinese population. *Journal of Endodontics*.

[16] Versteeg K. H., Sanderink G. C., van Ginkel F. C., van der Stelt P. F. (1997). Estimating distances on direct digital images and conventional radiographs. *Journal of American Dental Association*.

[17] Mohammadi Z., Giardino L., Palazzi F. (2013). Effect of sodium hypochlorite on the substantivity of chlorhexidine. *International Journal of Clinical Dentistry*.

[18] Lo Giudice G., Lizio A., Lo Giudice R. (2016). The effect of different cleaning protocols on post space: a SEM study. *International Journal of Dentistry*.

[19] Pinsky H. M., Dyda S., Pinsky R. W., Misch K. A., Sarament D. P. (2006). Accuracy of three-dimensional measurements using CBCT. *Dentomaxillofacial Radiology*.

[20] Lo Giudice G., Iannello G., Terranova A., Lo Giudice R., Pantaleo G., Cicciù M. (2015). Transcrestal sinus lift procedure approaching atrophic maxillary ridge: a 60-month clinical and radiological follow-up evaluation. *International Journal of Dentistry*.

[21] Patel S., Durack C., Abella F. (2014). European society of endodontology position statement: the use of CBCT in endodontics. *International Endodontic Journal*.

[22] Patel S., Durack C., Abella F., Shemesh H., Roig M., Lemberg K. (2015). Cone beam computed tomography in endodontics—a review. *International Endodontic Journal*.

[23] Bornstein M. M., Lauber R., Sendi P., von Arx T. (2011). Comparison of periapical radiography and limited cone-beam computed tomography in mandibular molars for analysis of anatomical landmarks before apical surgery. *Journal of Endodontics*.

[24] Altman D. G. (1991). *Practical Statistics for Medical Research*.

[25] Patel S., Wilson R., Dawood A., Foschi F., Mannocci F. (2012). The detection of periapical pathosis using digital periapical radiography and cone beam computed tomography-part 2: a 1-year post-treatment follow-up. *International Endodontic Journal*.

[26] Lo Giudice G., Matarese G., Lizio A. (2016). Invasive cervical resorption: a case series with 3-year follow-up. *International Journal of Periodontics and Restorative Dentistry*.

[27] Bender I. B., Seltzer S. (1961). Roentgenographic and direct observation of experimental lesions in bone. Part I. *Journal of American Dental Association*.

[28] Tyndall D. A., Rathore S. (2008). Cone-beam CT diagnostic applications: caries, periodontal bone assessment, and endodontic applications. *Dental Clinics of North America*.

[29] Vizzotto M. B., Silveira P. F., Arús N. A., Montagner F., Gomes B. P., da Silveira H. E. (2013). CBCT for the assessment of second mesiobuccal (MB2) canals in maxillary molar teeth: effect of voxel size and presence of root filling. *International Endodontic Journal*.

[30] Durack C., Patel S., Davies J., Wilson R., Mannocci F. (2011). Diagnostic accuracy of small volume cone beam computed tomography and intraoral periapical radiography for the detection of simulated external inflammatory root resorption. *International Endodontic Journal*.

[31] Cheung G. S., Wei W. L., McGrath C. (2013). Agreement between periapical radiographs and cone-beam computed tomography for assessment of periapical status of root filled molar teeth. *International Endodontic Journal*.

[32] Low K. M., Dula K., Bürgin W., von Arx T. (2008). Comparison of periapical radiography and limited cone-beam tomography in posterior maxillary teeth referred for apical surgery. *Journal of Endodontics*.

[33] Soğur E., Gröndahl H. G., Baksı B. G., Mert A. (2012). Does a combination of two radiographs increase accuracy in detecting acid-induced periapical lesions and does it approach the accuracy of cone-beam computed tomography scanning?. *Journal of Endodontics*.

[34] Venskutonis T., Daugela P., Strazdas M., Juodzbalys G. (2014). Accuracy of digital radiography and cone beam computed tomography on periapical radiolucency detection in endodontically treated teeth. *Journal of Oral and Maxillofacial Research*.

[35] Varshosaz M., Tavakoli M. A., Mostafavi M., Baghban A. A. (2010). Comparison of conventional radiography with cone beam computed tomography for detection of vertical root fractures: an in vitro study. *Journal of Oral Science*.

[36] Venskutonis T., Juodzbalys G., Nackaerts O., Mickevicienė L. (2013). Influence of voxel size on the diagnostic ability of cone-beam computed tomography to evaluate simulated root perforations. *Oral Radiology*.

[37] ICRP (2007). Publication 103: the 2007 recommendations of the international commission on radiological protection. *Annals of ICRP*.

[38] SEDENTEXCT (2012). *European Commission, Radiation Protection N 172: Cone Beam CT  for Dental and Maxillofacial Radiology. Evidence based Guidelines*.

